# Anxiety and Depression in Adolescents with Prelingual Hearing Loss: Prevalence and Risk Factors

**DOI:** 10.3390/jcm14217538

**Published:** 2025-10-24

**Authors:** Vijayalakshmi Easwar, Jason Gavrilis, Pelle Söderström, Teresa Ching, Greg Leigh, Vicky Zhang

**Affiliations:** 1 National Acoustic Laboratories, 16 University Ave, Macquarie Park, Sydney, NSW 2113, Australia; 2 Next Sense Institute, Macquarie School of Education, Macquarie University, 2 Gymnasium Road, Sydney, NSW 2113, Australia

**Keywords:** anxiety and depression, prelingual hearing loss, hearing aids, cochlear implants

## Abstract

**Objectives**: The aim of this study was to evaluate the prevalence of anxiety and depression symptoms and their predictors in 16–19-year-old adolescents with prelingual hearing loss (HL) who use spoken language. **Methods**: Self- and parent-reported symptoms were measured using RCADS-25 in 250 adolescents with HL (55.2% males; mean age = 17.1 years). A normal hearing (NH) peer group of 69 adolescents (56.5% males; mean age = 16.7 years) completed the self-reported RCADS-25. Key predictor variables included audiological factors, demographic factors, non-verbal IQ, language, communication, prosocial behaviour, and peer relations. **Results**: The proportion of adolescents with high self-reported anxiety was similar between HL and NH groups (~8%). Depression symptoms were more common in the HL group (11.2% self-reported, 15.8% parent-reported) than in the NH group (7.2%), but the difference was not statistically significant. Across informants, females had worse symptoms, but this association was no longer significant after accounting for communication difficulty. Among hearing aid users, higher prosocial behaviour was associated with fewer depression symptoms, while peer relations were a protective factor in cochlear implant users. Parent- and self-report congruence in symptom rating was modest (*r* = 0.56–0.68). Predictors of symptoms were consistent across informants, with parent happiness and socio-economic status additionally influencing parent-reported symptoms. Symptoms were unrelated to device type (hearing aid/cochlear implant), degree of hearing loss, or age at intervention. Adolescents with elevated symptoms also reported increased school absenteeism. **Conclusions**: Adolescents with HL reported anxiety at similar rates to NH peers but may have a higher prevalence of depression. Emotional well-being was influenced primarily by psychosocial and communication factors, not audiological characteristics.

## 1. Introduction

Children with hearing loss (HL) are known to be at increased risk for experiencing symptoms of anxiety and depression [1]. Anxiety and depression are two distinct but related internalising symptoms of psychopathology [2] and are among the most common psychiatric disorders in youth. These symptoms can have significant consequences across multiple aspects of life, including academic performance, career attainment, social functioning, and overall quality of life [3,4]. Therefore, a better understanding of the factors that increase vulnerability to, or protect against, these symptoms is essential for informing effective screening and intervention strategies. The purpose of the present study was to better understand the mental/emotional well-being of adolescents with prelingual hearing loss and those who use spoken language as their main mode of communication.

The prevalence of anxiety and depression symptoms has varied across studies and is influenced by numerous factors. Overall prevalence rates of mental health disorders in children with HL range between 18 and 77%, a wide range mainly arising from differences in demographic and communication characteristics of the study sample (e.g., severe HL, those who predominantly sign), sampling methods (e.g., self or parent-reported, or from medical records/diagnosed by psychiatrist), and criteria used for ascertaining clinically significant symptoms [5]. Deaf and hard of hearing youth were found to be at least 2–3 times more likely than their normal hearing (NH) peers to experience internalising symptoms [6]. Increased anxiety and phobia remained even after adjusting for child development, family, and school factors [7]. Reported odds of diagnosed depression have ranged between 2 and 2.78 in children and young adults with worse than moderate degree of HL [8]. If a longer time span is considered, ~26% have been found to experience depression at least once in their lifetime [9].

Internalising symptoms are reported to be influenced by certain HL characteristics, communication modality, child’s gender, communication abilities, and school attended (systematic review by Theunissen et al. [1]). A study that assessed children’s depression symptoms as a function of the age of HL onset and resolution found that, compared to children without HL, only children with late-onset HL (i.e., where HL started after 5 years of age) that did not resolve at 14 years of age had significantly higher odds of depression symptoms (adjusted OR = 3.21; 1.91–5.4; Butcher et al. [10]). Peer victimisation was found to be a contributing factor; children with HL that did not resolve had higher adjusted odds of peer victimisation, regardless of the age of HL onset [10].

In the case of communication modality, greater anxiety has been reported in signing deaf than hard of hearing, both being higher than the NH peers [9]. In the case of depression, although one study found that all children with HL experienced worse symptoms than their NH peers with minimal differences evident between those using signed and spoken language [9], another reported higher depression symptoms in those who signed compared to those who predominantly used oral language [11]. Females with HL are commonly reported to have higher anxiety and fears, as well as depression symptoms, than their male peers [6,12,13,14,15,16]. Interestingly, only in females were depression symptoms more strongly associated with peer victimisation [10].

The main communication-related predictor of internalising symptoms is reported to be the ability to make oneself understood in the family [17]. This finding is consistent with other studies that demonstrated the positive impact of stronger language and communication skills [18,19,20]. In fact, if communication skills were good, the increased risk of internalising was no longer evident even in those who used sign language [15]. Both internalising symptoms have been shown to be higher in children attending special schools than those who attended mainstream (ref. [6]; systematic review by Theunissen et al. [1]).

The influence of some factors continues to remain unclear. A factor with inconsistent evidence for its effect on internalising symptoms is the degree of HL. HL degree did not play a significant role in some studies [11,16,21] but did in others [6]. Another factor is the hearing device used (i.e., hearing aid [HA] vs. cochlear implant [CI]). Some studies have reported no difference between CI users and NH peers [22,23] and others have found no differences between HA or CI users [24]. On the contrary, some find fewer anxiety symptoms in CI users compared to those with HA [16]. Other factors include IQ and the presence of intellectual disabilities. While some show no association [16,25], some demonstrate that the presence of intellectual disabilities increases the risk of internalising symptoms [6]. These discrepancies may, in part, arise from study-specific inclusion/exclusion criteria (such as IQ exceeding the cut-off value or falling within the typical/normal range).

The studies reviewed here have significantly advanced our understanding of the mental well-being of children and youth with HL. It may be, however, that the findings of many of those studies are not generalisable to a contemporary population of adolescents with prelingual HL who use oral communication. Factors such as earlier HL identification through universal newborn hearing screening and the earlier provision of hearing technologies are likely to facilitate better levels of language and communication in current populations. Given the previously observed relationships between those abilities and both anxiety and depression, it may be that mental well-being outcomes are quite different in current populations relative to the outcomes found in older studies, or in those that have had exclusive criteria related to degree of HL or presence of additional disabilities. Another factor that may limit the generalisability of previous research is the variability in the instruments used to measure elements of mental well-being. Most previous studies have relied on measures based on either parent- or self-report but not used both. It is apparent that parent reports alone might under- or overestimate the behaviours of adolescents because they spend so little time with them through the day [26]. Additionally, it is well acknowledged that internalising symptoms are often difficult to observe and are more likely to go unnoticed by parents than externalising behaviours [27].

Given these considerations, the present study objectives were to: (i) evaluate the prevalence of self- and parent-reported anxiety and depressive symptoms in adolescents with long-term permanent HL using oral/spoken language, broadly representative of the current clinical population, and (ii) examine participant-specific and intervention-related factors associated with anxiety and depression symptom severity. Participant-specific factors included HL degree, communication difficulty/handicap, expressive social language use, non-verbal IQ, prosocial behaviour, peer relations, presence of additional disability, and socio-economic advantage. Intervention-related factors included device used and age at which the devices were fit. For parent-report measures, parent happiness was also included.

## 2. Materials and Methods

### 2.1. Participants

Adolescents with permanent HL enrolled in the Longitudinal Outcomes of Children with Hearing Impairment study [28] were invited to participate in this study. The primary eligibility criterion was based on parent/self-report of using spoken/oral language as the main method of communication (see Table 1 for number eligible). The use of sign language or mixed methods of communication was a criterion for exclusion. Matched in age, gender and socio-economic status to the cohort with HL, 69 adolescents with NH were invited for the study. Written consent was given by both the carer or adolescent, or either, depending on factors such as adolescent cognitive capacity or non-contact with carers. The study protocol was approved by the Hearing Australia Human Research Ethics Committee (AHHREC2019-2). Table 1 provides the demographic characteristics of the study cohort who completed the self-reported Revised Children’s Anxiety and Depression Scale (RCADS-25) [29]. Compared to the number of completed self-reported RCADS-25, there were slightly more parent-completed RCADS-25 in the HL cohort (6 more in the HA group, 1 more in the unaided group, 1 fewer for the bone conduction [BC] group for both anxiety and depression; 3 more for the depression subscale and 2 more for the anxiety subscale in the CI group).

### 2.2. Instruments

#### 2.2.1. Anxiety and Depression Symptoms

The 25-item Revised Child Anxiety and Depression Scale (RCADS-25) was used to identify symptoms of anxiety and depression [29]. The items in RCADS-25 are based on the Diagnostic and Statistical Manual of Mental Disorders—Fourth Edition [31], with two broad total subscales for anxiety and depression. The anxiety subscale comprises a range of anxiety symptoms, while the depression subscale measures symptoms of major depressive disorder (MDD). All items in RCADS-25 are rated on a four-point Likert scale from “Never” (0) to “Always” (4).

All participants completed the self-report version of RCADS-25, and for participants with HL, the primary carer additionally completed the parent-version of RCADS-25, answering questions about the participant. The items in both versions are identical, with the only difference being that the self-report asks questions from the first-person perspective (“I…”), while the parent-report asks questions in the third person (“My child…”). Both versions were administered via a custom-built survey in REDCap.

Raw RCADS-25 scores (Total Anxiety and Total Depression subscales) were transformed into t-scores according to normative age and sex data, with a mean of 50 and a standard deviation of 10. T-scores of 65 (1.5 SD above the mean) or higher were considered an indication of being at the borderline clinical threshold, whereas t-scores of 70 (2 SD above the mean) or higher indicate that the score is above the clinical threshold for depression or anxiety [29].

#### 2.2.2. Factors Associated with Anxiety and Depression Symptoms

The following questionnaires or standardised tests were used to assess different metrics, most of which were used as predictors.

(a) Communication difficulty: The ‘times’ subscale of the Self-Assessment of Communication—Adolescent (SAC-A) was used [32] to assess self-reported communication difficulty. This subscale asks six questions about difficulties hearing and understanding at different times, including when talking in groups of people and listening to the radio or TV. Each question in the subscale is scored from 1 (“almost never”) to 5 (“almost always”). Scores are summed, and higher summed scores indicate greater handicap. The ‘times’ subscale of the Significant Other Assessment of Communication—Adolescent (SOAC-A) [32] was used to assess the parent/carer-reported communication difficulty. SOAC-A is identical to SAC-A, except for asking the questions from the parent’s perspective of the child (e.g., “Is it hard for your child to…”). The internal consistency (Cronbach’s alpha) of SAC-A is 0.85, indicating acceptable internal consistency [33].
(b) Expressive language: The Communication Checklist—Self Report (CC-SR) [34] is a participant/self-reported measure of language structure, pragmatic skills, and social engagement. The language structure subscale used in the present study measures expressive use of grammar and language in social situations (e.g., leaving out or substituting sounds in words; mixing up words like “he” and “him”). For the purposes of scoring, responses were scored as numerical values between 0 (“less than once a week”) to 3 (“several times a day”) and converted to z-scores. For the parent-version, the Communication Checklist—Adult (CC-A) [35] was used. The items and scoring method are identical to the CC-SR, except for reporting in the third person (e.g., “S/he mixes up words that sound similar”). Higher z-scores indicate better language. The internal consistency of CC-A items (Cronbach’s alpha) ranges between 0.91 and 0.97. For CC-SR, the range is between 0.83 and 0.93, indicating acceptable internal consistency [34].
(c) Non-verbal IQ: The norm-referenced Test of Non-Verbal Intelligence (TONI-4) was used [36]. It is designed to require minimal language from both the administrator and the test taker. Index scores are calculated such that 100 is the mean, with a standard deviation of 15. TONI-4 has a Cronbach’s alpha of 0.96, suggesting high internal consistency [36].
(d) Prosocial behaviour: The prosocial subscale of the 25-item self- and parent-reported Strengths and Difficulties (SDQ) questionnaire [37,38] designed for children 11–17 years was used. The SDQ probes the frequency of behaviours related to prosocial behaviour (e.g., whether the person is considerate of other people’s feelings) on a three-point scale (“not true” (0), “somewhat true” (1), “certainly true” (2)). Scores were converted to z-scores using published norms [39]. Higher z-scores indicate better prosocial behaviour. The internal consistency (Cronbach’s alpha) of the self-reported prosocial behaviour scale is 0.65, suggesting acceptable internal consistency [38].
(e) Peer relations: The peer relations subscale of the SDQ that measures the child’s relationship to their peers was used. Higher z-scores indicate less difficulties. The internal consistency (Cronbach’s alpha) of the self-reported peer relations scale is 0.61, suggesting acceptable internal consistency [38].
(f) Additional disabilities: Participants with HL as well as their parents or carers were asked whether the adolescent had “any conditions, disabilities or special needs diagnosed” and responded by ticking boxes or entering any condition indicated as “Other”. For the regressions, the presence of any one additional disability was used as the predictor. Reported additional disabilities included autism spectrum disorder, cerebral palsy, intellectual/developmental disability, attention deficit hyperactivity disorder, attention deficit disorder, vision impairment, learning disability, craniofacial abnormality, and musculoskeletal disorders.
(g) Parent happiness: Parents/carers were asked “Overall, how satisfied are you with life as a whole these days?” and indicated their answer on a scale from 1 (“Not at all satisfied”) to 10 (“Completely satisfied”).
(h) School days off: To assess whether there was any association between the reported anxiety and depression symptoms and absenteeism, adolescents were asked to estimate the number of days they did not attend school over the past 12 months because of sickness.

With the exception of non-verbal IQ, all measures were completed by the participant/parent via a custom-built survey on REDCap. TONI-4 was administered by a trained speech pathologist or audiologist.

On average, self-report expressive language z-scores (CC-SR) were within 1 SD of the normative mean in both HL (mean, 1SD, min–max = 0.26, 1.3, −3.8–2.6) and NH groups (0.45, 1.1, −2.1–2.6). Corresponding parent-report scores (CC-A) in HL indicated average scores slightly below the normative mean (−1.1, 0.99, −4–0.1).

On average, the self-report SDQ subscale z-scores for peer relations as well as the prosocial subscale were within the normal range in HL (peer relations: −0.63, 1.2, −4.7–1.0; prosocial: 0.08, 1.1, −2.8–1.5). Parent-reported scores were similarly within 1 SD of the normative range (peer relations: −0.39, 1.1, −4.1–0.9; prosocial: −0.13, 1.13, −3.8–1.2).

Mean SAC scores were below 20%. This indicates “no disability/handicap” [40], suggesting low overall difficulty in self-reported communication in the HL cohort (16.3, 4.9, 5–30). Similarly, SOAC scores indicated low difficulty in the HL cohort (16.6, 4.6, 6–28).

### 2.3. Statistical Analysis

All statistical analyses were completed using R v4.4.2 [41]. To assess whether the RCADS-25 t-scores for anxiety and depression differed between adolescents with HL and NH, independent *t*-tests were used. To assess whether the proportion of adolescents with HL experienced higher anxiety/depression relative to NH peers, Chi-square tests were used.

To assess factors that influenced self- or parent-reported anxiety and depression symptoms, multiple linear regression was used. For both self- and parent-report, to assess whether symptoms differed by the device used, an initial regression analysis was carried out with factors common to both HA and CI users. Subsequently, additional regression analyses were performed with HA or CI users with device-specific factors. In this subsequent device-specific regression analysis, to ensure that significant predictors common to both CI and HA users were accounted for during device-specific regressions, they were included in the subsequent analyses. For the regression analysis, multiple imputation was used to handle missing independent variables in the data (average of 3–12.1% between self- and parent-report) with 20 imputations and then pooled via the *pool()* function from the *mice* package in R [42]. The association between self- and parent-reported symptoms was assessed using Pearson correlation. The association between the severity of anxiety/depression symptoms and days-off taken was assessed using Chi-square tests.

## 3. Results

### 3.1. Anxiety and Depression Symptoms of Adolescents with HL and NH

**Figure 1:**
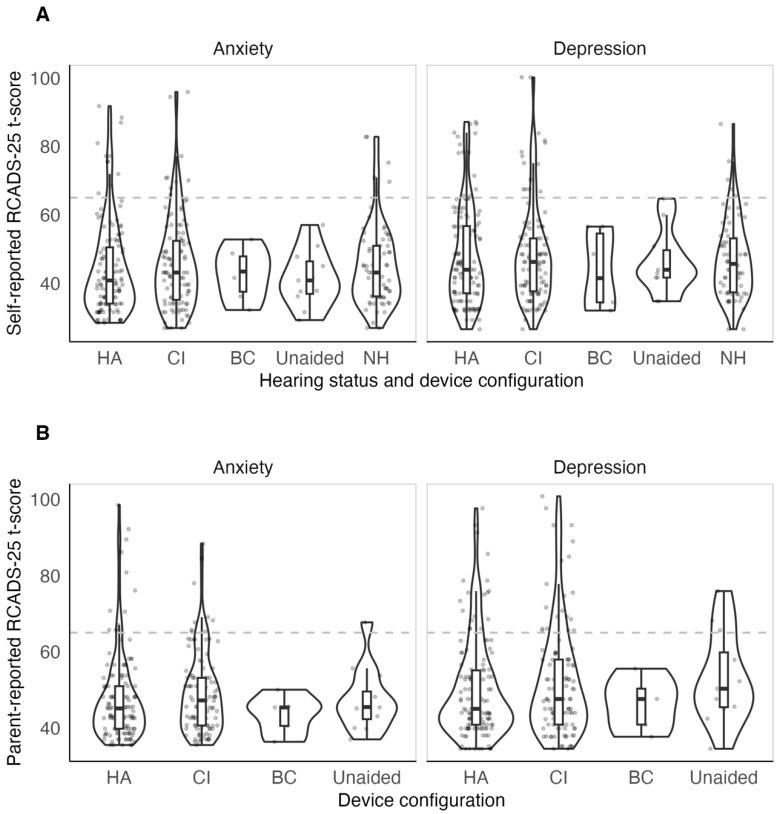
(**A**) t-scores from the self-report RCADS-25 as a function of hearing status and device configuration. (**B**) t-scores from the parent-report RCADS-25 as a function of hearing status and device configuration. Each unfilled circle represents one participant. The horizontal dashed line at 65 marks 1.5 SD above the normative mean. Scores below 65 are considered low severity while those above 65 are considered medium-high severity (clinical threshold for borderline clinical and clinical range, respectively). Panel B does not include NH participants because they did not have the parent-reported RCADS-25. HA = hearing aid, CI = cochlear implant, BC = bone conduction; NH = normal hearing.

Anxiety and depression subscale t-scores based on the device worn by children are shown in Figure 1. Children with bimodal configuration were combined with the CI group. Children with unilateral and bilateral devices were grouped together. Unlike the NH, CI and HA device users, self-reported t-scores of adolescents in the unaided and BC device user groups did not exceed the clinical cut-off of 65 (clinical borderline range).

Table 2 provides a summary of the t-scores per subscale and the proportion of adolescents with anxiety and depression symptoms in the typical and clinical range (t-score of ≥65) in both adolescents with HL and NH. Independent *t*-tests revealed no significant differences between the two groups in self-reported t-scores in both anxiety (*t* [118] = −0.27, *p* = 0.790) and depression (*t* [131] = 0.49, *p* = 0.622) subscales. Conclusions were unchanged even when unaided children were excluded.

The proportion of adolescents with high severity of self-reported anxiety was similar between HL (8%) and NH (7.2%) groups. The proportion of adolescents with high severity of self-reported depression was higher in HL (11.2%) compared to NH (7.2%); however, the tests comparing the proportions did not reach statistical significance (Anxiety: *X*^2^ [1] < 0.001, *p* = 0.999; Depression: *X*^2^ [1] = 0.53, *p* = 0.465). Among adolescents with HL, the proportion of adolescents with high severity of anxiety and depression was not statistically significant when reported by self and by their parent (Anxiety: *X*^2^ [1] < 0.01, *p* = 0.957; Depression: *X*^2^ [1] = 1.95, *p* = 0.163). The remainder of the study focuses on HA and CI users due to low numbers of unaided and BC device users.

### 3.2. Factors Associated with Self-Reported Anxiety Symptoms in HA and CI Users

Table 3 presents the results of linear regression analyses conducted separately for three groups: all groups combined, HA users only, and CI users only. When considering all groups together, higher levels of self-reported anxiety symptoms were significantly associated with being female and having lower expressive language abilities. In the analyses restricted to HA and CI users, higher self-reported anxiety symptoms were linked to lower expressive language abilities and greater communication difficulties, both based on self-report.

### 3.3. Factors Associated with Self-Reported Depression Symptoms in HA and CI Users

Table 4 provides the outcomes of the linear regression analyses carried out in all groups, HA users and CI users. With all groups combined, higher t-scores on the depression subscale were associated with being female, higher non-verbal IQ, lower SES, and lower expressive language abilities. Among HA users, higher depression t-scores were associated with lower expressive language abilities, worse prosocial behaviour, and increased communication difficulties. Among CI users, higher depression t-scores were associated with lower expressive language abilities, worse peer relations, and increased communication difficulties.

### 3.4. Association Between Parent and Self-Reported Anxiety and Depression Symptoms in HA and CI Users

**Figure 2:**
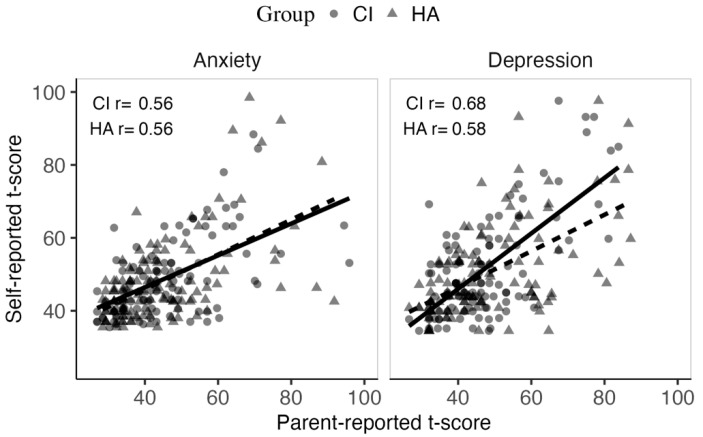
Association in RCADS-25 t-score between parent-report (horizontal axis) and self-report (vertical axis). Solid (CI group) and dashed (HA group) linear trends were both significant (*p* < 0.001). CI = cochlear implant; HA = hearing aid; r = correlation co-efficient.

Figure 2 illustrates the modest association in parent- and self-reported RCADS-25 t-scores. Table 5 provides the congruence in parent- and self-ratings in adolescents who had both parent- and self-ratings. While parent-rating coincides with self-ratings in the majority, in 11.01% (24/227) to 14.03% (32/228), either parent- or self-ratings indicated high anxiety or depression. This suggests that a multi-informant approach will be beneficial.

### 3.5. Factors Associated with Parent-Report Anxiety and Depression Symptoms

Table 6 provides the outcomes of the linear regression carried out to identify predictors associated with increased parent-reported anxiety in adolescents with HL. Higher parent-reported anxiety was associated with being female, lower SES, better prosocial behaviour, more peer problems, and increased communication difficulty. Higher parent-reported depression symptoms in adolescents were associated with the same factors as anxiety, with the exception of prosocial behaviour being non-significant and parent happiness being significant.

### 3.6. Relation Between Anxiety and Depression Symptoms and Days off School

**Figure 3:**
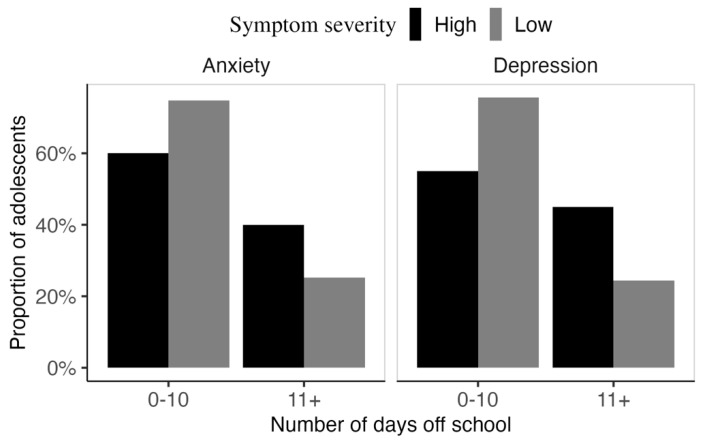
Association between number of school leave days and severity of self-reported anxiety and depression symptoms.

Figure 3 shows the proportion of adolescents with HL reporting ≤10 vs >10 days of school absence, categorised by severity of self-reported anxiety and depression symptoms. Data were available for 217 adolescents. In both panels, a higher number of days off was more commonly observed among adolescents reporting higher symptom severity. However, Chi-square analysis revealed no significant difference in days off taken by severity of anxiety symptoms (*X*^2^ [1] = 1.57, *p* = 0.210). In contrast, there was a significant association between depression severity and days off school (*X*^2^ [1] = 3.99, *p* = 0.045). Similar patterns were observed when the analysis was confined to CI or HA users (Anxiety: *X*^2^ [1] = 1.79, *p* = 0.180; Depression: *X*^2^ [1] = 4.45, *p* = 0.035).

## 4. Discussion

The present study investigated anxiety and depression symptoms in adolescents with prelingual HL who used spoken language as the main mode of communication. Children were diagnosed and received their first hearing device by 3 years of age, and the majority attended mainstream school.

### 4.1. Prevalence of Clinically Relevant Anxiety and Depression Levels Did Not Vary Significantly Between Adolescents with and Without HL

Contrary to most earlier studies that included only significant degrees of HL and/or sign language users, group-level comparisons in the present study revealed no statistically significant differences in mean anxiety or depression symptoms between adolescents with HL and NH. Although twice the proportion of adolescents with HL reported depression symptoms in the clinical range compared to NH peers, this difference did not reach statistical significance (Table 2). These findings highlight the heterogeneity of mental health outcomes within the HL population and underscore the need for individual-level screenings/assessments.

The lack of statistically significant differences in internalising symptoms between adolescents with and without HL (Table 2, Table 3 and Table 4, Figure 1) coincides with some studies that have predominantly assessed children with CI and/or HA [22,23]. The lack of differences between adolescents wearing different devices (CI vs. HA) also agrees with some previous studies [24]. Previous studies that found differences between HL and NH children, and between CI and HA users, likely varied in sample characteristics such as communication abilities and/or modality, influencing interactions with peers and family. The present study confirms that early HL diagnosis and intervention may be effective in minimising the difference between NH and HL in anxiety and depression symptoms, likely facilitated by achieving better communication abilities and well-supported families through subsidised services such as those provided in Australia.

A novel aspect of this study is the use of a clinically representative sample, which included adolescents who used bone conduction devices or had very limited use of devices and essentially being unaided. When disaggregated by device configuration, unaided and bone conduction device users had notably lower self-reported symptoms, with scores not exceeding clinical thresholds (Figure 1). However, given the small sample sizes in these groups, caution is warranted in interpretation. The sample also included children with additional disabilities although this factor was also not a significant predictor for the internalising symptoms. This could have been because the impact was captured by other outcomes such as expressive language, communication difficulty, and peer relations/prosocial behaviour.

### 4.2. Influential Factors of Mental Well-Being in Adolescents: Gender, Expressive Language, Communication Difficulty and Psychosocial Behaviour

When self-reported, adolescent females had 3.1–5.9 times higher odds than males of showing clinically significant anxiety symptoms, across both HL and NH cohorts. The analogous odds ratios for depression symptoms were 3.5–5.9. When expressive language and communication difficulty were accounted for, gender was no longer a predictive factor for either self-reported anxiety or depression (Table 3 and Table 4), possibly suggesting that these communication and language-related challenges mediate the observed gender difference in emotional well-being. The increased risk of depression symptoms among adolescent girls is evident in the general population as well [43]. The relation with increased communication/language difficulties is also consistent with previous work in children [18,19,20].

Depression symptoms were significantly associated with parent-reported peer relations for adolescents with CI and HA, and with self-reported peer relations for those with CI (Table 4 and Table 6). The likely bidirectional association between poor peer relations and increased risk of depression symptoms [44] aligns with prior research [45]. Self-reported depression symptoms were associated with prosocial behaviour in HA users but not CI users (Table 4). Similarly, no associations were found in parent-reported depression symptoms (Table 6). Reduced prosocial behaviour being a risk factor for depressive symptoms in adolescents has been reported in the general population [46] and in HL groups, however, prosocial behaviour may not always be protective. In fact, one of the reasons for greater depression risk in female adolescents is thought to be heightened prosociality that may be at a personal cost [43]. This could have contributed to inconsistent associations.

Self-reported anxiety was not associated with peer or prosocial behaviour; however, both factors were associated with parent-reported anxiety (Table 3 and Table 6). The differences probably arise from informant differences [26], wherein parents, observing social withdrawal or reduced prosociality, may interpret these as markers of anxiety, and that may differ from how adolescents perceive it.

Parent-reported outcomes were influenced by a similar constellation of factors: female gender, lower socio-economic status, and greater communication difficulty. Additionally, parental perceptions of their own happiness emerged as a significant predictor, suggesting that the family-emotional climate may influence how parents perceive their child’s mental health. This is consistent with findings in the general population [26].

Neither age of intervention nor the degree of HL was a significant predictor in the current cross-sectional analysis, as has been observed in other studies [11,47]. This could indicate that communication difficulties and language abilities at 16–19 years are more important in explaining variance in mental health symptoms at the same age. A similar explanation could be provided for non-verbal IQ being a significant (or near-significant) influencer of internalising symptoms only when communication difficulties were not yet included in the regression (Table 3 and Table 4).

### 4.3. A Multi-Informant Approach Is Favourable to Assess Mental Health Challenges in Adolescents

Interestingly, while parent- and self-ratings of symptoms were significantly correlated, a notable proportion (9.7–15.4%) of adolescents showed clinical symptoms on only one of the two informant reports. Further, the factors associated with increased parent-perceived anxiety or depression symptoms were not identical to analogous relationships in self-report symptoms (Table 3 and Table 4 vs. Table 6). Together, the present study continues to emphasise the value of a multi-informant approach when evaluating emotional well-being.

### 4.4. Clinical Implications

Characterising the incidence of mental health disorders is critical, as unmanaged symptoms can lead to immediate issues such as school avoidance and reduced academic engagement (Figure 3), as well as increased risk of self-harm, which is reported to be higher in children with HL (OR = 1.41; 95% CI: 1.12–1.78; Butcher et al. [10]). In the long-term, earlier identification and management may reduce probability of mood, learning, academic, and conduct problems frequently associated with internalising symptoms [48].

The present study used a standard questionnaire with normative data that may help identify children/adolescents needing referrals to non-audiology professionals. Similar to recommendations provided by Cejas et al. [49] for use in otology clinics, periodic use in audiology clinics may help audiologists make appropriate referrals and assist with improving more holistic care.

The present study provided factors that were both protective and detrimental to internalising symptoms based on cross-sectional factors. Future work could focus on predictive factors in a longitudinal design to identify early hearing and communication symptoms that influence later mental health challenges so that psychopathology could be effectively prevented.

### 4.5. Strengths and Limitations

There are strengths and limitations associated with the present study. Major strengths include the use of a large, representative sample, clinically relevant cut-offs for depression and anxiety scores, the inclusion of both self- and parent-reported data, as well as the minimisation of bias through using multiple imputation rather than complete-case analysis to account for missing data.

While RCADS-25 has representative questions from the comprehensive 47-item version [50] for the different types of anxiety (covering, e.g., panic disorder and separation anxiety disorder), different types of anxiety could not be reliably quantified because cut-off scores for clinical interpretation are lacking, with the current RCADS-25 focusing on broad anxiety and depression subscales rather than interpretable subscales [29]. These areas could be probed in future work.

The present study examined participant-related factors assessed concurrently with anxiety and depression symptoms. Future research should investigate earlier-developing participant and family-level risk factors to inform preventive strategies and support long-term mental well-being.

It is also possible that many adolescents with HL currently receive help for mental health concerns. This may have minimised the group difference. Unfortunately, we did not capture this information for the present study.

Various hormones can influence depression and anxiety symptoms in children and adolescents (ref. [51]; see Luo, Dashti, Sawyer & Vijayakumar [52] for a review). However, we did not capture hormonal data in the present study and thus could not account for its potential influence on the results.

## 5. Conclusions

Using a large and clinically representative study sample of adolescents with permanent prelingual HL and using both self- and parent-report, the present study establishes that, on average, anxiety and depression symptoms reported during adolescence (16–19 years) are comparable between those with and without HL. At the individual level, the proportion experiencing significant anxiety symptoms was similar between groups. Although significant depression symptoms were reported in 11 to 15.8% of adolescents with HL compared to 7% in their NH peers, this difference was not statistically significant. Together, anxiety symptoms were greater in females, as well as in those with worse expressive language abilities and increased communication difficulty. Depression symptoms were additionally greater in those with higher IQ and greater socio-economic disadvantage. Increased prosocial behaviour reduced the risk of depression symptoms in hearing aid users similarly to better peer relations in cochlear implant users. Neither anxiety nor depression symptoms were associated with device being used, HL severity, or age of intervention/device fitting. The positive association between increased depression severity and more frequent absenteeism from school illustrates the immediate impact of mental well-being.

## Figures and Tables

**Table 1:** Demographic characteristics of adolescents who completed the RCADS-25 questionnaire (self-report version). Socio-economic status was assessed through quartiles of the Index of Relative Socio-economic Advantage and Disadvantage (IRSAD) from the Australian Bureau of Statistics [30]. HA = hearing aid, CI = cochlear implant, BC = bone conduction, NH = normal hearing.

		HA	CI	BC	Unaided	NH
Number		127	106	6	11	69
Age at test (years)	mean (SD; min–max)	17.14 (0.7; 16–19.3)	16.96 (0.7; 16–19.2)	16.9 (0.5; 16.3–17.6)	17.17 (0.7; 16.4–18.9)	16.81 (0.6; 16–18)
Gender	Female	54 (42.5%)	52 (49.1%)	3 (50%)	3 (27.3%)	30 (43.5%)
	Male	73 (57.5%)	54 (50.9%)	3 (50%)	8 (72.7%)	39 (56.5%)
Non-verbal IQ	mean (SD; min–max)	97.73 (10.1; 71–122)	96.66 (11.9; 70–122)	96.25 (11.2; 85–111)	88.62 (10.6; 70–107)	100.46 (10.3; 83–122)
Better ear 4-freq average (dB HL)	mean (SD; min–max)	51.01 (15.1; 23.8–105)	112.51 (23.2; 30–125)	63.12 (5; 53.8–67.5)	31.82 (11.4; 20–51.2)	19 (0; 19–19)
Better ear HL degree	Normal	0 (0%)	0 (0%)	0 (0%)	0 (0%)	69 (100%)
	Mild	33 (26%)	2 (1.9%)	0 (0%)	7 (63.6%)	0 (0%)
	Moderate	84 (66.1%)	7 (6.6%)	6 (100%)	4 (36.4%)	0 (0%)
	Severe	10 (7.9%)	97 (91.5%)	0 (0%)	0 (0%)	0 (0%)
Age at first hearing device (months)	mean (SD; min–max)	10.68 (10.2; 1.2–35.8)	8.62 (8.9; 0.9–34.3)	2.23 (1.2; 1.2–3.9)	13.11 (10.1; 2.5–36.4)	NA
Age at first CI (months)	mean (SD; min–max)	NA	37.68 (39.6; 5–184)	NA	NA	NA
Device configuration	Unilateral	4 (3.1%)	7 (6.6%)	1 (16.7%)	NA	NA
	Bilateral	123 (96.9%)	77 (72.6%)	5 (83.3%)	NA	NA
	Bimodal	NA	22 (20.8%)	NA	NA	NA
Additional Disabilities	Yes	54 (42.5%)	37 (34.9%)	4 (66.7%)	4 (36.4%)	0 (0%)
	No	73 (57.5%)	69 (65.1%)	2 (33.3%)	7 (63.6%)	69 (100%)
Device use all day	Yes	88 (69.3%)	93 (87.7%)	6 (100%)	0 (0%)	0 (0%)
	No	35 (27.6%)	12 (11.3%)	0 (0%)	7 (63.6%)	0 (0%)
	Declined to answer	4 (3.1%)	1 (0.9%)	0 (0%)	4 (36.4%)	NA
IRSAD quartile	1	12 (9.4%)	11 (10.4%)	1 (16.7%)	2 (18.2%)	10 (14.5%)
	2	22 (17.3%)	19 (17.9%)	0 (0%)	2 (18.2%)	14 (20.3%)
	3	29 (22.8%)	20 (18.9%)	5 (83.3%)	2 (18.2%)	18 (26.1%)
	4 (most advantage)	64 (50.4%)	56 (52.8%)	0 (0%)	5 (45.5%)	27 (39.1%)
School type	Distance education	1 (0.8%)	0 (0%)	0 (0%)	0 (0%)	0 (0%)
	Homeschool	1 (0.8%)	0 (0%)	0 (0%)	0 (0%)	0 (0%)
	Mainstream	109 (85.8%)	92 (86.8%)	6 (100%)	11 (100%)	69 (100%)
	Special education	4 (3.1%)	3 (2.8%)	0 (0%)	0 (0%)	0 (0%)
	Not at school	12 (9.4%)	9 (8.5%)	0 (0%)	0 (0%)	0 (0%)
	Declined to answer	0 (0%)	2 (1.9%)	0 (0%)	0 (0%)	0 (0%)

**Table 2:** Number and proportion of participants within each severity category of the anxiety and depression subscales of RCADS-25. *N* = total sample size; *n* = subset sample size; SD = standard deviation.

		Anxiety				Depression			
Informant	Group	*N*	Mean (SD)	High Severity, *n* (%)	Low Severity, *n* (%)	*N*	Mean (SD)	High Severity, *n* (%)	Low Severity, *n* (%)
Self	HL	250	44.78 (13.4)	20 (8%)	230 (92%)	250	47.85 (14.3)	28 (11.2%)	222 (88.8%)
Self	NH	69	45.23 (12.1)	5 (7.2%)	64 (92.8%)	69	47.03 (11.6)	5 (7.2%)	64 (92.8%)
Parent	HL	258	48.19 (11)	22 (8.5%)	236 (91.5%)	259	51 (13.6)	41 (15.8%)	218 (84.2%)

**Table 3:** Output of multiple regression analyses predicting self-reported anxiety t-scores. Independent variables in bold indicate statistically significant predictors. HA = hearing aid, CI = cochlear implant, NH = normal hearing, SE = standard error; df = degrees of freedom; BE 4freq PTA = better ear 4 frequency pure tone average.

NH, HA, CI (*n* = 302)	Estimate (SE)	t (df)	*p* Value
(Intercept)	39.32 (6.8)	5.78 (225.7)	<0.001
Group: HA (reference NH)	−0.48 (1.7)	−0.28 (291.9)	0.779
Group: CI (reference NH)	−1.65 (1.8)	−0.92 (292.0)	0.359
**Gender: Male (reference female)**	−5.57 (1.3)	−4.18 (290.5)	<0.001
Non-verbal IQ	1.23 (0.7)	1.82 (218.8)	0.070
SES	−0.35 (0.7)	−0.52 (290.5)	0.603
**Expressive language**	−5.19 (0.6)	−9.03 (283.8)	<0.001
Adjusted R^2^			27.30%
HA (*n* = 127)	Estimate (SE)	t (df)	*p* value
(Intercept)	46.38 (6.3)	7.35 (114.9)	<0.001
Gender: Male (reference female)	−1.93 (2.3)	−0.83 (114.9)	0.406
Additional Disabilities: Yes (reference none)	−1.52 (2.2)	−0.70 (115.4)	0.484
**Expressive language**	−3.75 (1.1)	−3.49 (112.0)	0.001
Age at first HA fit	−1.75 (1.1)	−1.64 (115.5)	0.104
Prosocial behaviour	−1.45 (1.1)	−1.33 (112.7)	0.186
Peer relations	−1.94 (1.0)	−1.94 (112.5)	0.055
**Communication difficulty**	0.57 (0.3)	2.13 (114.6)	0.036
BE 4freq PTA	−1.01 (0.7)	−1.36 (115.4)	0.176
Adjusted R^2^			31.70%
CI (*n* = 106)	Estimate (SE)	t (df)	*p* value
(Intercept)	16.59 (10.6)	1.57 (94.4)	0.120
Gender: Male (reference female)	−4.01 (2.3)	−1.77 (94.5)	0.079
Additional Disabilities: Yes (reference none)	−3.16 (2.4)	−1.31 (94.5)	0.192
**Expressive language**	−3.10 (0.9)	−3.38 (93.2)	0.001
Age at first CI	2.20 (1.5)	1.48 (94.3)	0.142
Prosocial behaviour	1.62 (1.0)	1.58 (93.9)	0.118
Peer relations	−1.22 (1.0)	−1.25 (94.4)	0.214
**Communication difficulty**	0.89 (0.3)	3.40 (94.6)	0.001
BE 4freq PTA	0.77 (0.6)	1.38 (94.2)	0.170
Adjusted R^2^			31.70%

**Table 4:** Output of multiple regression analyses predicting self-reported depression t-scores. Independent variables in bold indicate statistically significant predictors. HA = hearing aid, CI = cochlear implant, NH = normal hearing, SE = Standard error; df = degrees of freedom; BE 4freq PTA = better ear 4 frequency pure tone average; SES = socio-economic status.

NH, HA, CI (*n* = 302)	Estimate (SE)	t (df)	*p* Value
(Intercept)	37.78 (7.1)	5.35 (266.4)	<0.001
Group: HA (reference NH)	1.56 (1.8)	0.85 (291.7)	0.397
Group: CI (reference NH)	−0.10 (1.9)	−0.05 (292.3)	0.959
**Gender: Male (reference female)**	−4.85 (1.4)	−3.40 (290.1)	0.001
**Non-verbal IQ**	1.87 (0.7)	2.68 (265.2)	0.008
**SES**	−1.59 (0.7)	−2.23 (291.4)	0.026
**Expressive language**	−4.89 (0.6)	−7.99 (287.0)	<0.001
Adjusted R^2^			24.20%
HA (*n* = 127)	Estimate (SE)	t (df)	*p* value
(Intercept)	31.82 (12.5)	2.54 (108.1)	0.012
Gender: Male (reference female)	0.61 (2.4)	0.25 (112.0)	0.801
Non-verbal IQ	2.03 (1.2)	1.74 (106.9)	0.085
Additional Disabilities: Yes (reference none)	−0.48 (2.2)	−0.21 (112.8)	0.831
SES	−1.03 (1.2)	−0.88 (111.8)	0.379
**Expressive language**	−3.53 (1.2)	−3.02 (109.2)	0.003
Age at first HA fit	−1.45 (1.3)	−1.11 (113.2)	0.271
**Prosocial behaviour**	−3.44 (1.1)	−3.05 (111.1)	0.003
Peer relations	−2.14 (1.1)	−1.98 (110.2)	0.050
**Communication difficulty**	0.62 (0.3)	2.17 (112.5)	0.032
BE 4freq PTA	−1.32 (0.8)	−1.71 (113.3)	0.090
Adjusted R^2^			35.30%
CI (*n* = 106)	Estimate (SE)	t (df)	*p* value
(Intercept)	14.07 (16.0)	0.88 (89.8)	0.380
Gender: Male (reference female)	−4.47 (2.5)	−1.79 (92.4)	0.077
Non-verbal IQ	2.02 (1.1)	1.91 (85.3)	0.059
Additional Disabilities: Yes (reference none)	−0.90 (2.6)	−0.34 (91.6)	0.735
SES	−2.03 (1.2)	−1.74 (92.9)	0.086
**Expressive language**	−2.48 (1.1)	−2.28 (87.9)	0.025
Age at first CI	1.24 (1.6)	0.76 (92.3)	0.450
Prosocial behaviour	−1.15 (1.1)	−1.02 (91.8)	0.309
**Peer relations**	−2.16 (1.1)	−2.03 (92.3)	0.045
**Communication difficulty**	0.81 (0.3)	2.81 (92.5)	0.006
BE 4freq PTA	0.36 (0.6)	0.59 (92.3)	0.556
Adjusted R^2^			36.30%

**Table 5:** Congruence between parent- and self-rating. Each cell provides the number of adolescents in each congruence pattern, the total available responses, and the proportion in parenthesis. A t-score of <65 was considered low severity. CI = cochlear implant; HA = hearing aid.

Subscale	Group	Low—Both	High—Both	High—Parent Only	High—Self Only
Anxiety	CI	89/104 (85.6)	3/104 (2.9)	6/104 (5.8)	6/104 (5.8)
	HA	106/123 (86.2)	5/123 (4.1)	6/123 (4.9)	6/123 (4.9)
Depression	CI	82/105 (78.1)	10/105 (9.5)	9/105 (8.6)	4/105 (3.8)
	HA	98/123 (79.7)	6/123 (4.9)	11/123 (8.9)	8/123 (6.5)

**Table 6:** Output of multiple regression analyses predicting parent-reported anxiety and depression t-scores. Independent variables in bold indicate statistically significant predictors. HA = hearing aid, CI = cochlear implant, NH = normal hearing, SE = standard error; df = degrees of freedom; BE 4freq PTA = better ear 4 frequency pure tone average; SES = socio-economic status.

Parent-rated Anxiety			
HA, CI (*n* = 241)	Estimate (SE)	t (df)	*p* value
(Intercept)	43.85 (8.0)	5.47 (187.3)	<0.001
Group: CI (reference HA)	−0.17 (2.3)	−0.07 (226.4)	0.941
**Gender: Male (reference female)**	−3.95 (1.2)	−3.16 (226.0)	0.002
Non-verbal IQ	0.55 (0.6)	0.86 (148.8)	0.392
Additional Disability: Yes (reference None)	1.43 (1.3)	1.09 (226.7)	0.277
**SES**	−1.71 (0.6)	−2.74 (226.3)	0.007
Parent happiness	−0.78 (0.4)	−1.77 (213.7)	0.078
Parent-rated Expressive language	0.11 (0.7)	0.16 (207.0)	0.875
**Parent-rated prosocial behaviour**	1.55 (0.6)	2.62 (225.8)	0.009
**Parent rated peer relations**	−3.65 (0.7)	−5.40 (226.6)	<0.001
**Parent-rated communication difficulties**	0.64 (0.1)	4.48 (222.3)	<0.001
BE 4freq PTA	0.04(0.3)	0.11 (226.4)	0.911
Adjusted R^2^			32.02%
			
Parent-rated Depression			
HA, CI (*n* = 242)	Estimate (SE)	t (df)	*p* value
(Intercept)	43.17 (9.6)	4.49 (170.5)	<0.001
Group: CI (reference HA)	1.89 (2.7)	0.70 (226.6)	0.486
**Gender: Male (reference female)**	−4.16 (1.5)	−2.83 (226.2)	0.005
Non-verbal IQ	0.97 (0.8)	1.22 (109.9)	0.225
Additional Disability: Yes (reference None)	2.17 (1.5)	1.40 (227.4)	0.162
**SES**	−1.71 (0.7)	−2.33 (225.9)	0.021
**Parent happiness**	−1.20 (0.5)	−2.38 (227.4)	0.018
Parent-rated Expressive language	0.93 (0.8)	1.10 (201.4)	0.273
Parent-rated prosocial behaviour	−0.60 (0.7)	−0.86 (227.0)	0.389
**Parent rated peer relations**	−4.37 (0.8)	−5.51 (227.3)	<0.001
**Parent-rated communication difficulties**	0.85 (0.2)	5.01 (216.4)	<0.001
BE 4freq PTA	−0.19 (0.4)	−0.50 (226.7)	0.62
Adjusted R^2^			38.10%

## Data Availability

The original contributions presented in this study are included in the article. Further inquiries can be directed to the corresponding author.
